# Cause-Specific Mortality among Infants in a Randomized Controlled Trial of Azithromycin Compared to Placebo for Prevention of Mortality

**DOI:** 10.4269/ajtmh.24-0186

**Published:** 2024-09-24

**Authors:** Mamadou Ouattara, Ali Sié, Mamadou Bountogo, Valentin Boudo, Thierry Ouedraogo, Clarisse Dah, Elodie Lebas, Huiyu Hu, Aimee Lansdale, Ian Fetterman, Benjamin F. Arnold, Thomas M. Lietman, Catherine E. Oldenburg

**Affiliations:** ^1^ Centre de Recherche en Santé de Nouna, Nouna, Burkina Faso;; ^2^ Francis I. Proctor Foundation, University of California, San Francisco, San Francisco, California;; ^3^ Department of Epidemiology & Biostatistics, University of California, San Francisco, San Francisco, California;; ^4^ Department of Ophthalmology, University of California, San Francisco, San Francisco, California

## Abstract

Although community randomized trials have found a reduction in all-cause child mortality in communities receiving mass azithromycin distribution compared with placebo, individually randomized trials have not found similar protective effects. If a direct effect of azithromycin for prevention of child mortality exists, it is likely due to reduction in infectious mortality. Here, we assessed cause-specific mortality in a large randomized controlled trial of azithromycin administered during well-infant visits in Burkina Faso for prevention of mortality. Among 32,877 enrolled infants, the most common causes of death by 6 months of age were malaria, acute respiratory infections, and diarrheal disease. We found no evidence of a difference in the distribution of cause of death by randomized treatment assignment (*P* = 0.42) or in any infectious-specific cause of death. The results of this analysis are consistent with no direct effect of azithromycin on infant mortality when administered during well-infant visits.

## INTRODUCTION

Biannual mass drug administration with single-dose azithromycin has been shown to reduce all-cause child mortality among children aged 1–59 months in the MORDOR and CHAT trials.^1^^,^^2^ Subgroup analysis of the MORDOR trial suggested that the strongest effects of azithromycin may be in infants, who have the highest mortality rates.^1^ Studies evaluating distribution of azithromycin to infants have included both mass distribution (e.g., treating all infants in the community at a given time) and targeted (e.g., treating infants on an individual basis as they come into the clinic) approaches.^3^^,^^4^ A large randomized controlled trial of azithromycin distribution at the time of well-child visits in Burkina Faso demonstrated no difference in all-cause mortality among infants receiving azithromycin compared with placebo.^4^ Similarly, an individually randomized trial of neonatal azithromycin (NAITRE) found no evidence of a difference in all-cause infant mortality compared with placebo.^5^

The mechanism of action behind azithromycin for prevention of mortality in children is not clear, but presumably involves reduction in clinical or subclinical infections. Protection may be via direct or indirect effects. If indirect, mass treatment of an entire community may be necessary to reduce transmission of pathogens.^6^ In MORDOR, cause-specific mortality due to infectious disease including malaria, dysentery, pneumonia, and meningitis was reduced in communities receiving azithromycin compared with placebo.^7^ In individually randomized trials, any effect of azithromycin would be due to direct effects of the intervention. In NAITRE, mortality attributed to malaria was approximately half as common in neonates receiving azithromycin compared with those receiving placebo.^8^ Here, we report cause-specific mortality outcomes in an individually randomized trial of azithromycin integrated into well-child visits in Burkina Faso, including vaccination visits, that found overall no difference in all-cause mortality.^4^ We hypothesized that azithromycin would reduce infectious causes of mortality in infants, with no effect on noninfectious causes.

## MATERIALS AND METHODS

### Trial methods.

Complete methods for the trial have been previously described.^4^^,^^9^ In brief, infants aged 5–12 weeks were randomized 1:1 to a single, oral dose of azithromycin (20 mg/kg) or matching placebo. Participants were enrolled from September 2019 until October 2022. Infants were recruited during well-infant visits in primary healthcare clinics or via community-based outreach in Nouna, Banfora, and Karankasso-Vigue, Burkina Faso.^10^ Infants were eligible if they were 5–12 weeks of age at enrollment and had no documented allergies to macrolides and if their family was planning to remain in the trial area for the 6-month study period. Participants, caregivers, trial staff, and investigators were masked to the infant’s randomized treatment assignment. The primary endpoint for the trial was all-cause mortality by 6 months of age. The trial was reviewed and approved by the Institutional Review Board at the University of California, San Francisco and the Comité d’Ethique pour la Recherche en Santé in Ouagadougou, Burkina Faso. Written informed consent was obtained from the guardian of each enrolled infant.

### Verbal Autopsy Methods.

Each infant who died during the study was eligible for a verbal autopsy using the WHO 2016 verbal autopsy tool for children aged 4 weeks to 11 years. Verbal autopsies were performed after a 3-month mourning period. Verbal autopsy data were collected electronically using a custom-built application for a mobile phone. Cause of death was assigned using InSilicoVA in R, an algorithm that automatically assigns cause of death based on responses to the verbal autopsy instrument.^11^ We considered the cause of death assigned by the algorithm as being the most likely cause of death based on participant responses to be the cause of death for analysis purposes.

## STATISTICAL ANALYSES

We evaluated the overall distribution of cause of death as assigned by verbal autopsy among infants who died by randomized treatment assignment using a Fisher’s exact test. Cause-specific mortality was analyzed using binomial regression with a complementary log-log link to estimate the relative hazard of each cause of mortality separately between the randomized treatment groups. Treatment group was the sole predictor in these models. *P*-values were calculated via permutation test with 10,000 replications. All analyses were conducted in R (The R Foundation for Statistical Computing, Vienna, Austria).

## RESULTS

Of 32,877 infants enrolled in the trial, 157 died and were included in the primary analysis. Infants were an average of 6.6 weeks of age in the azithromycin arm and 6.7 weeks in the placebo arm at enrollment, and 49% were female in both arms (Supplemental Table 1). There was no evidence of a difference in mortality between infants receiving azithromycin or placebo in the primary analysis.^4^ Of 157 deaths in the trial, verbal autopsy results were available for 136 (87%; Supplemental Figure 1), including 72 (88%) in the azithromycin group and 64 (85%) in the placebo group. Overall, there was no difference in the distribution of cause of death by randomized treatment assignment (*P* = 0.42) or in any specific cause of death (Table 1). The most common cause of death was malaria, with 17 deaths in the azithromycin arm and 21 in the placebo arm (hazards ratio [HR]: 0.81; 95% CI: 0.43–1.53). The second- and third-most common causes of death were acute respiratory infections (azithromycin: 17, placebo: 20; HR: 0.85; 95% CI: 0.44–1.62) and diarrhea (azithromycin: 18, placebo: 10; HR: 1.80; 95% CI: 0.83–3.89).

**Table 1:** Cause-specific mortality among infants randomized to azithromycin (*N* = 15,734) or placebo (*N* = 15,701)

Cause of Death	*n* with Event (%)		HR (95% CI)	*P*-Value
	Azithromycin	Placebo		
Diarrheal Disease	18 (0.11)	10 (0.06)	1.80 (0.83–3.89)	0.15
Malaria	17 (0.11)	21 (0.13)	0.81 (0.43–1.53)	0.51
Acute Respiratory Infection	17 (0.11)	20 (0.13)	0.85 (0.44–1.62)	0.56
Other Infectious Disease	10 (0.06)	6 (0.04)	1.66 (0.60–4.58)	0.40
Meningitis and Encephalitis	8 (0.05)	3 (0.02)	2.66 (0.71–10.03)	0.20
Other Noninfectious Disease	2 (0.01)	3 (0.02%)	0.67 (0.11–3.98)	0.38
Accident	1 (0.01)	1 (0.01)	1.00 (0.06–15.95)	0.81

HR = hazard ratio.

## DISCUSSION

Overall, we found no evidence of a difference in cause-specific mortality by 6 months of age among infants who received a single, oral dose of azithromycin compared with placebo. A previous study of mass distribution of azithromycin found reduced infectious mortality in children living in communities receiving azithromycin compared with placebo in Niger.^7^ The most common causes of child mortality in settings such as Niger and Burkina Faso are infectious. Reductions in all-cause mortality have been observed when entire communities are treated with azithromycin via mass distribution (e.g., in community randomized trials), but not when individuals are treated (e.g., in individually randomized trials).^1^^,^^2^^,^^4^^,^^5^ For infectious causes of mortality for which azithromycin may be expected to have some activity, such as acute respiratory tract infections or diarrhea, a lack of effect of azithromycin may provide additional evidence that protection against mortality is not primarily via direct effect on treated individuals. Treatment of all children in a given community at the same time may reduce transmission of some pathogens that cause mortality. When children are treated on an individual basis, they may become rapidly reinfected by other children with whom they interact, limiting any protective effects.

Although the exact mechanism of azithromycin for reducing child mortality is unknown, it is likely multifactorial via reduction in infectious disease. Infectious causes of mortality, including malaria, were reduced in the Niger site of the MORDOR trial in communities receiving azithromycin compared with placebo.^7^ Similarly, a reduction in malaria parasitemia was observed in the same study.^12^ The present study found no evidence of an effect of individual azithromycin on malaria mortality, and previous individually randomized studies have failed to find evidence of an effect of azithromycin on malaria parasitemia.^13^^,^^14^ Azithromycin has weak antimalarial properties and is not effective as monotherapy for treating malaria.^15^ It is possible that mass azithromycin distribution to an entire community reduces some malaria transmission such that overall community-level prevalence is reduced but that the effect of azithromycin on malaria at the individual level is insufficient for any effect. Azithromycin is active against many pathogens that cause pneumonia and diarrhea, and mass distribution of azithromycin has been shown to reduce mortality due to pneumonia and diarrhea as assigned by verbal autopsy.^7^ Although genomic studies of individually randomized trials have shown a reduction in *Campylobacter* spp.,^16^ there is little evidence of the effect of individual dosing of azithromycin for pneumonia and diarrhea. It is possible that a similar pattern as that previously observed for malaria exists for other common infectious causes of mortality.

This study had several limitations to consider. Overall, mortality was lower than anticipated than during the planning of the trial, thus limiting statistical power for cause-specific mortality estimates. Sample size calculations were based on all-cause mortality (the primary endpoint) and assumed an all-cause mortality probability of 4%.^4^ This analysis was stratified by cause of death, further reducing statistical power. As a result, CIs were wide. Verbal autopsy has well-known limitations in its sensitivity and specificity for determining cause of death at an individual level.^17^^,^^18^ Methods such as minimally invasive tissue sampling would likely have yielded more accurate causes of death; however, these methods require substantially more resources and were not feasible in the present study. Future studies evaluating the effect of azithromycin on cause-specific mortality could conduct the trial in sites with existing mortality surveillance. Verbal autopsies assign a single most likely cause of death, thus potentially losing information about contributing etiology. Although the algorithm assigning cause of death was run with masking of each infant’s randomized treatment group, it is possible that this loss of information masked differences in cause of death between arms. All deaths in the study were identified via study personnel, including those that occurred within the health system. Although medical records may provide additional insights into cause of death for those that occurred within the health system, we were unable to link children enrolled in the trial to medical record data. Finally, these results may not be generalizable to higher mortality settings or areas with a different distribution of cause of death.

This analysis of verbal autopsy data of a large randomized controlled trial of azithromycin for prevention of infant mortality found no evidence of a difference in cause-specific mortality at 6 months of age among infants receiving a single, oral dose of azithromycin compared with placebo. These results do not provide evidence of a direct effect of azithromycin when administered to infants for prevention of mortality by 6 months of age.

## Supplemental Materials

10.4269/ajtmh.24-0186 Supplemental Materials
