# Supplementary material for: Cause-Specific Mortality among Infants in a Randomized Controlled Trial of Azithromycin Compared to Placebo for Prevention of Mortality
Source: Am J Trop Med Hyg. 2024 Sep 24;111(6):1353–5. doi: 10.4269/ajtmh.24-0186 (PMC11619516; doi:10.4269/ajtmh.24-0186)

Supplemental Table 1. Baseline characteristics of infants enrolled in a trial of azithromycin for prevention of infant mortality by treatment group.

	**Azithromycin N= 16416**	**Placebo N= 16461**
**Age in weeks, median (IQR)**	6.6 (5.0 to 8.7)	6.7 (5.1 to 8.9)
**Female sex, N (%)**	8045 (49.0%)	8136 (49.4%)

Supplemental Figure 1. Study flow diagram for infants in a trial of azithromycin for prevention of infant mortality with cause of death assessment by verbal autopsy

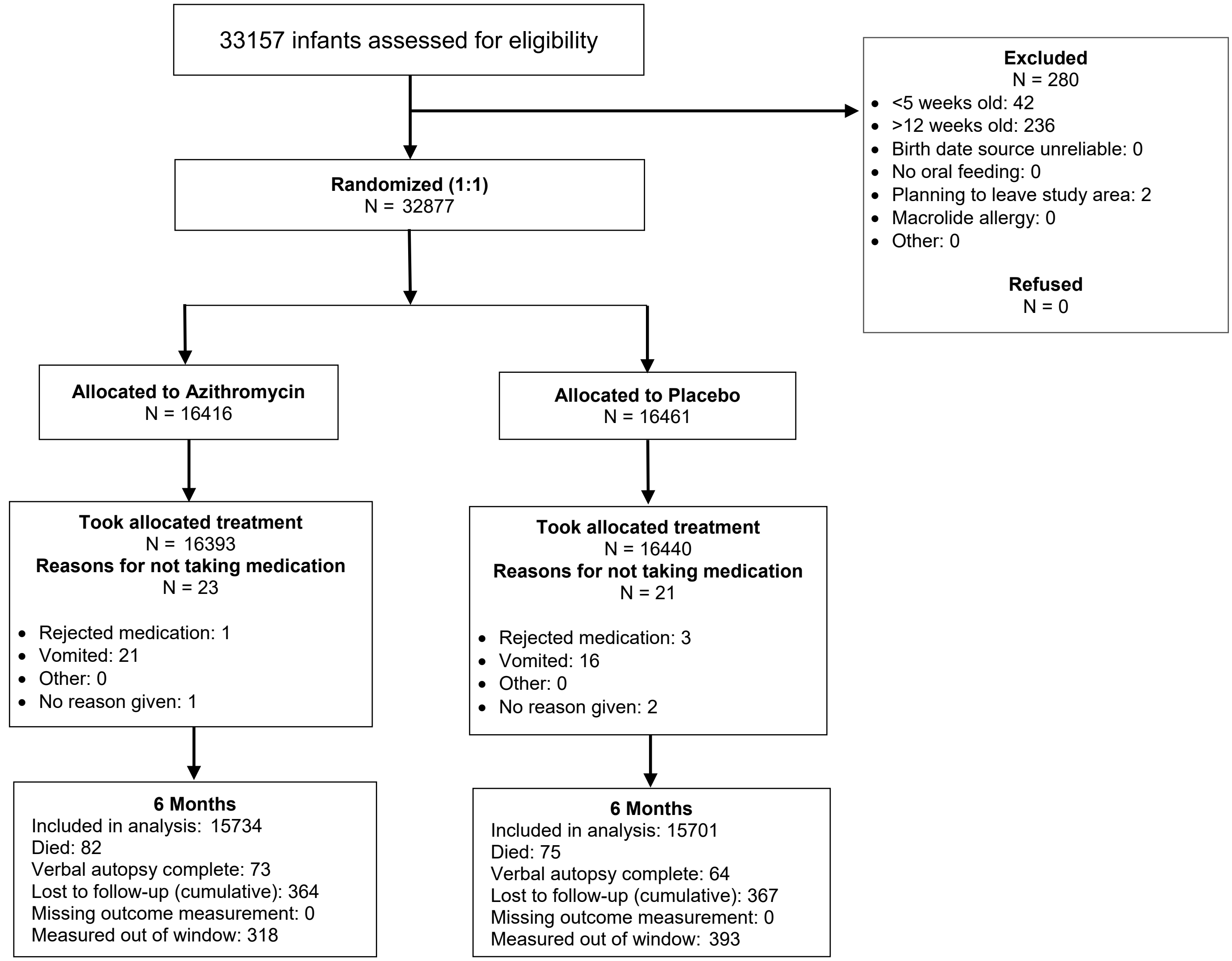

Supplement: Supplemental Materials [file tpmd240186.SD1.pdf]
